# Crimean-Congo Hemorrhagic Fever, Herat Province, Afghanistan, 2017

**DOI:** 10.3201/eid2508.181491

**Published:** 2019-08

**Authors:** Aziz-ur-Rahman Niazi, Mohammad Jawed Jawad, Ahmad Amirnajad, Peter A. Durr, David T. Williams

**Affiliations:** Herat University, Herat, Afghanistan (A.-u.-R. Niazi, M.J. Jawad);; Department of Public Health, Herat (A. Amirnajad); CSIRO,; Australian Animal Health Laboratory, Geelong, Victoria, Australia (P.A. Durr, D.T. Williams)

**Keywords:** Crimean-Congo hemorrhagic fever, Crimean-Congo hemorrhagic fever virus, CCHF, CCHFV, Afghanistan, clinical symptomatology, epidemiology, viruses, ticks, vector-borne infections

## Abstract

We studied the clinical and epidemiologic features of an outbreak of Crimean-Congo hemorrhagic fever in Herat Province, Afghanistan. The study comprised 63 patients hospitalized in 2017. The overall case-fatality rate was 22.2%; fatal outcome was significantly associated with a negative IgM test result, longer prothrombin time, and nausea.

Crimean-Congo hemorrhagic fever (CCHF) is a geographically widespread tickborne disease caused by the CCHF virus (genus *Orthonairovirus*, family *Nairoviridae*). In humans, CCHF is associated with a case-fatality rate (CFR) of 5%–50% (*1*) and is considered a major public health threat (*2*).

CCHF cases were first reported in Afghanistan in 1998; no additional cases were reported until 2007 (*3*). During 2007–2016, the Afghanistan Ministry of Public Health documented 478 cases, of which Herat Province accounted for 263 (55.0%) (*4*). In 2017, an unusual increase in CCHF cases occurred in Afghanistan, mostly in Herat Province (Appendix Figures 1, 2). We analyzed the clinical and epidemiologic features of this outbreak.

A descriptive case series study at Herat Regional Hospital during January–December 2017 was undertaken. Clinical and epidemiologic features of all confirmed and probable CCHF cases were recorded. The Human Ethics Committee of Herat University approved the study protocol (approval #0317).

The first recorded case in this study occurred in a 90-year-old male farmer who visited Herat Regional Hospital on May 5. Later, more patients sought care for acute febrile syndrome matching the World Health Organization CCHF case definition (*5*). A total of 64 patients sought care for CCHF signs and symptoms over a 6-month period, of whom 1 did not consent to hospitalization and left the hospital without medical consultation.

Patients’ venous blood samples were examined for levels of leukocytes, thrombocytes, aspartate aminotransferase, alanine aminotransferase, prothrombin time (PT), and activated partial thromboplastin time. Samples were also tested for CCHV IgM (VectoCrimea-CHF-IgM ELISA, Vector-Best, https://www.vector-best.ru) or viral RNA (RealStar CCHFV RT-PCR Kit, Altona Diagnostics, https://www.altona-diagnostics.com). We tested for an association among epidemiologic, clinical, and laboratory variables and clinical outcome for each patient (i.e., death or recovery) using Fisher exact test and a confirmatory multivariate logistic regression within R v3.5.0 (https://www.r-project.org).

Of the 63 patients, 32 were both IgM and PCR positive. Thirty-one patients were IgM-negative (Appendix Table 1); however, because of a positive PCR result (26 patients) or CCHF-specific clinical features (5 patients), these patients were included in this study. Thirty-eight (60.3%) patients were male (Appendix Table 2). Overall mean (+ SD) age was 35.4 (+ 20.0) years (range 9–90 years). Most (69.8%) patients were 11–40 years of age. The occupational groups most often affected were housewives (23 [36.5%] patients) and farmers (14 [22.2%]). Butchers accounted for 7 (11.1%) and shepherds for 3 (4.8%) cases; the remaining 16 (25.4%) patients had other occupations. Eighteen (28.6%) patients lived in the city, and 45 (71.4%) lived in rural areas. Three (4.8%) patients stated a history of tick bite, and 60 (95.2%) had prior contact with livestock or animal tissues.

The most frequent clinical manifestations were fever, headache, and myalgia, and the most common hemorrhagic manifestations were ecchymosis, epistaxis, and hemoptysis. At admission, all patients had thrombocytopenia, and 62 (98.4%) had leukopenia. Aspartate aminotransferase and alanine aminotransferase levels were elevated in 43 (68.3%) patients. PT time was longer than normal in 29 (46.0%) and activated partial thromboplastin in 23 (36.5%) patients (Table).

**Table:** Laboratory findings and clinical and hemorrhagic features for patients hospitalized with Crimean-Congo hemorrhagic fever, Herat Province, Afghanistan, 2017*

Characteristic	No. (%) patients, N = 63
Laboratory	
Thrombocytopenia†	63 (100.0)
Leukopenia	62 (98.4)
AST‡	43 (68.3)
ALT§	43 (68.3)
Longer PT	29 (46.0)
Longer aPTT	23 (36.5)
Clinical features	
Fever	63 (100.0)
Headache	59 (93.7)
Myalgia	53 (84.1)
Fatigue	51 (81.0)
Abdominal pain	17 (27.0)
Nausea	15 (23.8)
Vomiting	9 (14.3)

Type of hemorrhage	
Ecchymosis	42 (66.7)
Epistaxis	36 (57.1)
Hemoptysis	12 (19.1)
Hematemesis	10 (15.9)
Melana	7 (11.1)
Petechia	4 (6.4)
Gum bleeding	3 (4.8)

*ALT, alanine aminotransferase; aPTT, activated partial thromboplastin time; AST, aspartate aminotransferase; PT, prothrombin time. †<50 × 10^9^/L (reference 150–450 × 10^9^/L) (*6*). ‡>200 U/L (reference 5–40 U/L) (*7*). §>150 U/L (reference 7–56 U/L) (*7*).

All cases occurred during May–October; cases peaked during June–September (Appendix Figure 2). The average time (+ SD) between onset of clinical manifestations and patients’ referral to hospital was 4.9 (+ 1.6) days; (range 0–9 days. The overall CFR was 22.2% (14/63 patients); the CFR for men was 18.4% (7/38) and for women, 28.0% (7/25). Fatal outcome was significantly associated with a negative IgM test result (p = 0.016), longer PT (p = 0.038), and nausea (p = 0.015).

A previous study established that CCHF patients who die rarely mount a detectable IgM response, and laboratory diagnosis should therefore include reverse transcription PCR (*8*). A positive association of death with longer PT also has been previously described (*9*). A new finding from this study was the association between fatal outcome and nausea.

Our findings are important for persons in Afghanistan, especially in Herat Province, because the study identified demographic variables (age and occupation) that can be further investigated by a risk factor study. Our findings also are important for persons traveling to Herat Province. Only 1 CCHF case has thus far been reported in a tourist returning home from northwestern Afghanistan, where the disease was acquired (*10*). Our findings can also be used to refine the CCHF case definition for improved clinical awareness in Afghanistan.

Our study demonstrates that Herat Province remains the endemic focus of CCHF in Afghanistan, and the number of cases is increasing over time. Control and mitigation measures implemented for CCHF in Herat have not been successful in containing this fatal disease. Considering the major social and economic consequences and the health burden CCHF places on the community, alternative or enhanced public health measures, including improved surveillance and risk communication, are necessary to control CCHF in Herat and neighboring provinces. Our findings might serve as a template and reference for future CCHF surveillance activities in this region.

Appendix Additional case and location data for study of Crimean-Congo hemorrhagic fever, Herat Province, Afghanistan, 2017.
