# Supplementary material for: Crimean-Congo Hemorrhagic Fever, Herat Province, Afghanistan, 2017
Source: Emerg Infect Dis. 2019 Aug;25(8):1596–8. doi: 10.3201/eid2508.181491 (PMC6649324; doi:10.3201/eid2508.181491)
Supplement: Appendix — Additional case and location data for study of Crimean-Congo hemorrhagic fever, Herat Province, Afghanistan, 2017. [file 18-1491-Techapp-s1.pdf]

Article DOI: https://doi.org/10.3201/eid2508.181491

Crimean-Congo Hemorrhagic Fever, Herat Province, Afghanistan, 2017

Appendix

Appendix Table 1. Laboratory confirmation for 63 Crimean-Congo hemorrhagic fever cases, Herat Province, Afghanistan, 2017

	IgM		
PCR result	Positive	Negative	Case category
Positive	32	26	Confirmed
Negative	0	5	Probable

Appendix Table 2. Demographic characteristics of 63 patients hospitalized with Crimean-Congo hemorrhagic fever, Herat Province, Afghanistan, 2017

Characteristic	No. patients (%)
Sex	
M	38 (60.3)
F	25 (39.7)
Age group, y†	
0–10	1 (1.6)
11–20	19 (30.2)
21–30	16 (25.4)
31–40	9 (14.3)
41–50	5 (8.0)
51–60	5 (8.0)
61–70	5 (8.0)
71–80	1 (1.6)
81–90	2 (3.2)
Occupation	
Housewife	23 (36.5)
Farmer	14 (22.2)
Butcher	7 (11.1)
Laborer	6 (9.5)
Shepherd	3 (4.8)
Unemployed	3 (4.8)
Waiter	2 (3.2)
Engineer	1 (1.6)
Student	1 (1.6)
Driver	1 (1.6)
Security guard	1 (1.6)
Shopkeeper	1 (1.6)
Living environment	
Urban	18 (28.6)
Rural	45 (71.4)
Type of exposure	
History of tick bites	3 (4.8)
Contact with livestock and infected tissue	60 (95.2)

*Information about sex, age, occupation, living environment and exposure was recorded for all patients.
†Overall mean age ± SD: 35.4 ± 20.0.

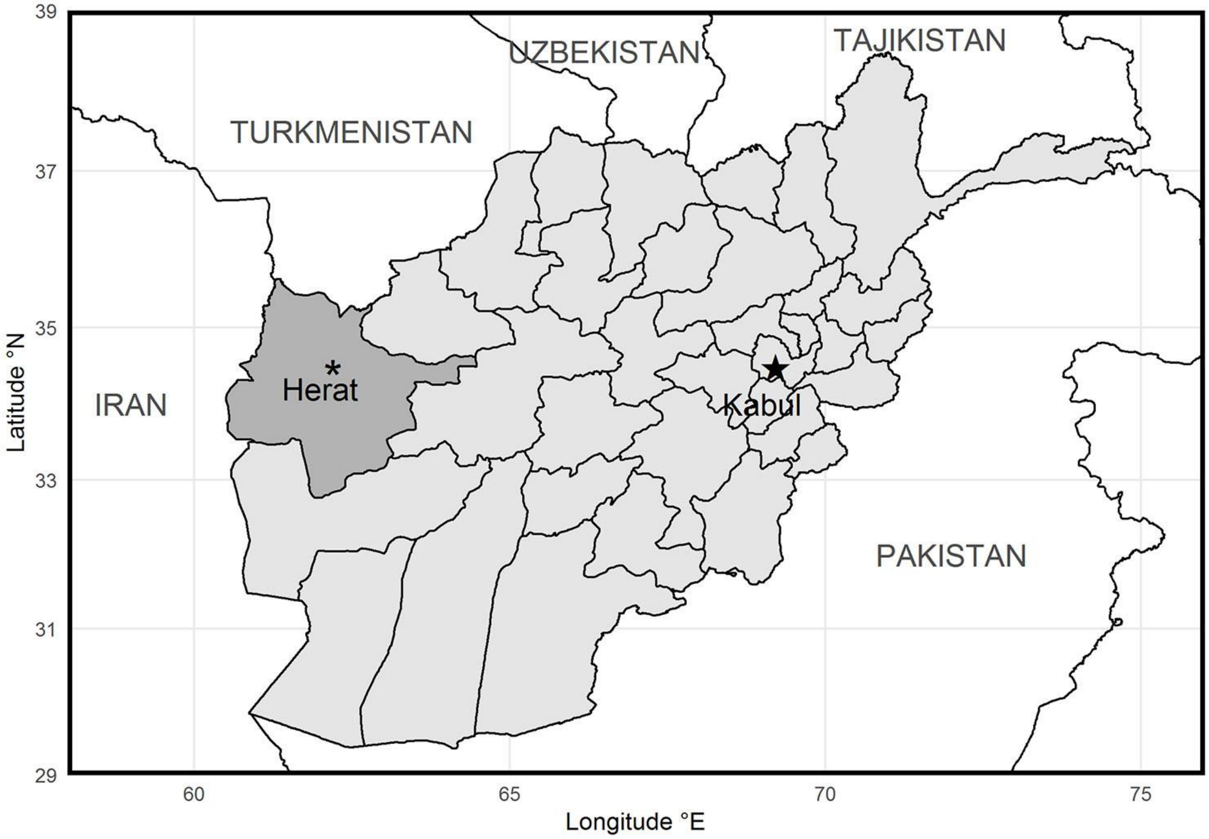

Appendix Figure 1. Location of Herat province (dark shading) in the west of Afghanistan (light shading) and surrounding countries.

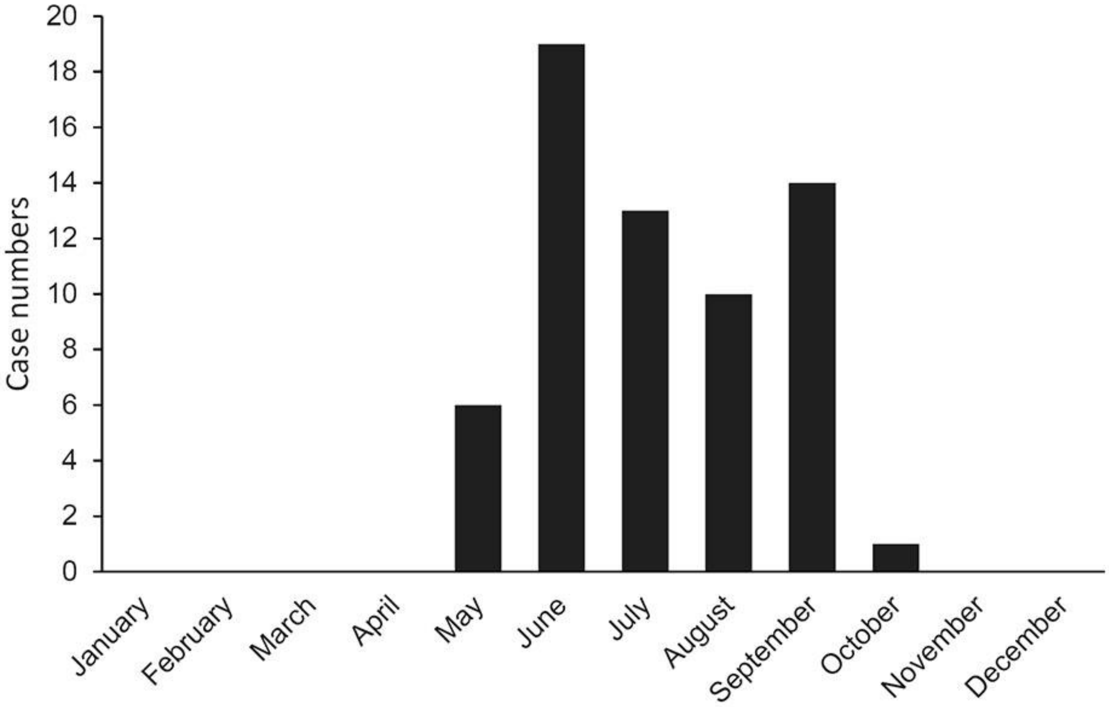

Appendix Figure 2. Number of new cases of Crimean-Congo hemorrhagic fever, Herat province, Afghanistan, 2017. Findings are based on notifications to the Disease Early Warning System of the Afghanistan Ministry of Public Health.
